# Magnolol and honokiol exert a synergistic anti-tumor effect through autophagy and apoptosis in human glioblastomas

**DOI:** 10.18632/oncotarget.8674

**Published:** 2016-04-11

**Authors:** Yu-Chen Cheng, Dueng-Yuan Hueng, Hua-Yin Huang, Jang-Yi Chen, Ying Chen

**Affiliations:** ^1^ Graduate Institute of Life Science, National Defense Medical Center, Taipei, Taiwan; ^2^ Department of Neurological Surgery, Tri-Service General Hospital, National Defense Medical Center, Taipei, Taiwan; ^3^ Department of Biochemistry, National Defense Medical Center, Taipei, Taiwan; ^4^ Department of Biology and Anatomy, National Defense Medical Center, Taipei, Taiwan

**Keywords:** honokiol, magnolol, autophagy, apoptosis, glioblastoma

## Abstract

Glioblastoma (GBM) is a malignant brain tumor associated with a high mortality rate. The aim of this study is to investigate the synergistic effects of honokiol (Hono) and magnolol (Mag), extracted from *Magnolia officinalis*, on cytotoxicity and inhibition of human GBM tumor progression in cellular and animal models. In comparison with Hono or Mag alone, co-treatment with Hono and Mag (Hono-Mag) decreased cyclin A, D1 and cyclin-dependent kinase 2, 4, 6 significantly, leading to cell cycle arrest in U87MG and LN229 human glioma cells. In addition, phosphorylated phosphoinositide 3-kinase (p-PI3K), p-Akt, and Ki67 were decreased after Hono-Mag treatment, showing proliferation inhibition. Hono-Mag treatment also reduced p-p38 and p-JNK but elevated p-ERK expression. Besides, Hono-Mag treatment induced autophagy and intrinsic and extrinsic apoptosis. Both ERK and autophagy inhibitors enhanced Hono-Mag-induced apoptosis in LN229 cells, indicating a rescuer role of ERK. In human GBM orthotopic xenograft model, the Hono-Mag treatment inhibited the tumor progression and induced apoptosis more efficiently than Temozolomide, Hono, or Mag group. In conclusion, the Hono-Mag exerts a synergistic anti-tumor effect by inhibiting cell proliferation and inducing autophagy and apoptosis in human GBM cells. The Hono-Mag may be applied as an adjuvant therapy to improve the therapeutic efficacy of GBM treatment.

## INTRODUCTION

Glioblastomas, also known as glioblastoma multiforme (GBM), are tumors usually found in the cerebral hemispheres of the brain. GBM is composed of poorly differentiated neoplastic astrocytes that exhibit aggressive proliferation and invasive properties [1, 2]. It is generally difficult to identify prognostic factors associated with tumors, and 95% of patients die within 3 months after diagnosis without therapy [3]. Although there has been considerable progress in the treatment of GBM over the past 20 years, the median survival of glioblastoma patients is still less than 15 months [4, 5]. The blood-brain barrier (BBB) and the blood-cerebrospinal fluid barrier impede the effects of both chemotherapy and target therapies. Therefore, the development of suitable drugs that can cross the BBB and act as adjuvant drugs to improve the efficacy of surgery and chemotherapy is important for the treatment of GBM.

Honokiol (Hono) and magnolol (Mag) are extracted from the root and stem bark of *Magnolia officinalis*. These two compounds are often used in traditional herbal medicine in East Asia for the treatment of gastrointestinal complaints, anxiety, thrombotic stroke, and nervous system dysfunction [6]. Hono or Mag treatments not only induce apoptosis but also suppress cell proliferation through cell cycle arrest and up-regulating the expression of pro-apoptosis molecules in human glioma cells [7–12]. Treatment with Mag promotes p21/Cip1 and p27/ Kip1 expression and inhibits proliferation in the human U373 cell line *in vitro* and *in vivo* [8]. Mag also induces autophagy and inhibits cell migration and invasion in human PC3 cells [13]. In recent studies, Hono treatment was found to significantly enhance reactive oxygen species (ROS) production, decrease mitochondrial membrane potential, release cytochrome c, and activate caspase-9 and caspase-3, which leads to apoptosis in human glioblastoma cells [11, 12]. Hono treatment blocks the phosphoinositide 3-kinase (PI3K)/mammalian target of rapamycin (mTOR) pathway-mediated immunoresistance of glioma without inhibiting critical proinflammatory T cell functions [14]. In addition, Hono effectively inhibits U87MG invasion and tumor growth in a human U251 xenograft glioma model [15, 16]. In prostate cancer, robust autophagy is triggered by Hono or Mag treatment, which inhibits angiogenesis and causes cell death in human umbilical cord vein endothelial cells and PC-3 cells [13, 17]. Recently, both Hono and Mag were reported being able to cross the BBB and inhibit cancer cell progression; however, the synergistic effect and mechanisms of Hono and Mag in the inhibition of proliferation and induction of cell death in glioma cancer cells have not been elucidated.

To investigate the synergistic effect of these two compounds in GBM treatment, we examined anti-tumor effects both *in vitro* and *in vivo*. The combination treatment of Hono with Mag (Hono-Mag) greatly limited the proliferation and survival of human GBM cells. Tumor size was also substantially decreased after Hono-Mag treatment in an orthotopic xenograft animal model. The synergistic effect of Hono-Mag treatment strongly inhibited glioma tumor growth through the autophagy formation and apoptosis pathways. In summary, the Hono-Mag combination treatment is more effective and has more potential in glioma treatment than treatment with Hono or Mag alone.

## RESULTS

### Hono and mag reduced the survival rate of human glioblastoma cells *in vitro*

**Figure 1:**
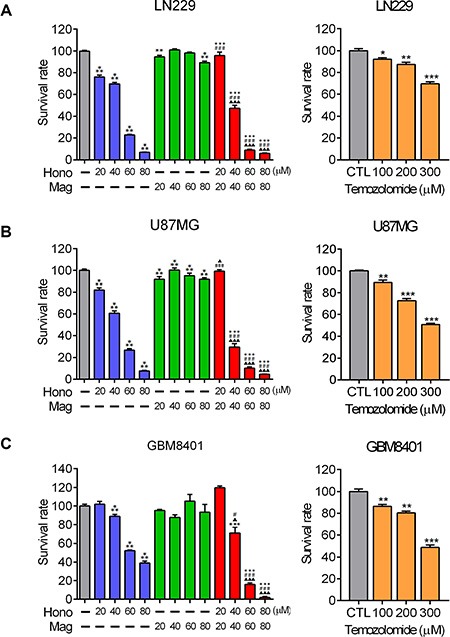
The effects of Hono, Mag and Hono-Mag combination treatment on cell viability (**A**) LN229 glioma cells, (**B**) U87MG glioma cells, and (**C**) GBM8401 glioma cells were treated with DMSO or 20, 40, 60, or 80 μM of Hono, Mag or Hono-Mag combination for 24 hours. After treatment, the survival rate was analyzed using MTT tests. The right panels showed TMZ-treated glioma cells which were regarded as a positive control. *p < 0.05; **p < 0.01; ***p < 0.001 compared to the control group. ^#^*p* < 0.05; ^##^*p* < 0.01; ^###^*p* < 0.001 compared to the same concentration of Hono-treated group. ^▲^*p* < 0.05; ^▲▲^*p* < 0.01; ^▲▲▲^*p* < 0.001 compared to the same concentration of Mag-treated group.

To determine the effect of Hono and Mag on the proliferation of human glioblastoma cells, the survival rates of LN229, U87MG and GBM8401 were assessed using MTT assays. The glioma chemotherapy drug TMZ was applied as a positive control. The survival rates of LN229, U87MG and GBM8401 were decreased in the concentration of 50 μM and above by TMZ treatment (Figure 1A, 1B, 1C). In the presence of 40 μM Hono for 24 hours, the survival rates of Hono-treated U87MG and LN229 cells were significantly reduced by more than 30% over control cells (Figure 1A, 1B). The survival rate of GBM8401 was also reduced by 10% after treatment with 40 μM Hono treatment for 24 hours (Figure 1C). In contrast, GBM cells treatment with Mag showed less decreased on the survival rate. However, when Hono was combined with Mag treatment at 40 μM for 24 hours, the survival rate remarkably decreased by over 50% in LN229 cells and 70% in U87MG cells compared with control. In addition, the survival rate of GBM8401 cells decreased by over 30% under the same combined treatment compared with control. The combination treatment of Hono and Mag at 60 μM and 80 μM substantially decreased the proliferation and survival rates of LN229, U87MG and GBM8401 cells as compared to the control group, Hono-alone group, or Mag-alone group.

### Hono and mag arrested the cell cycle and decreased the expression of cell cycle-related proteins

**Figure 2:**
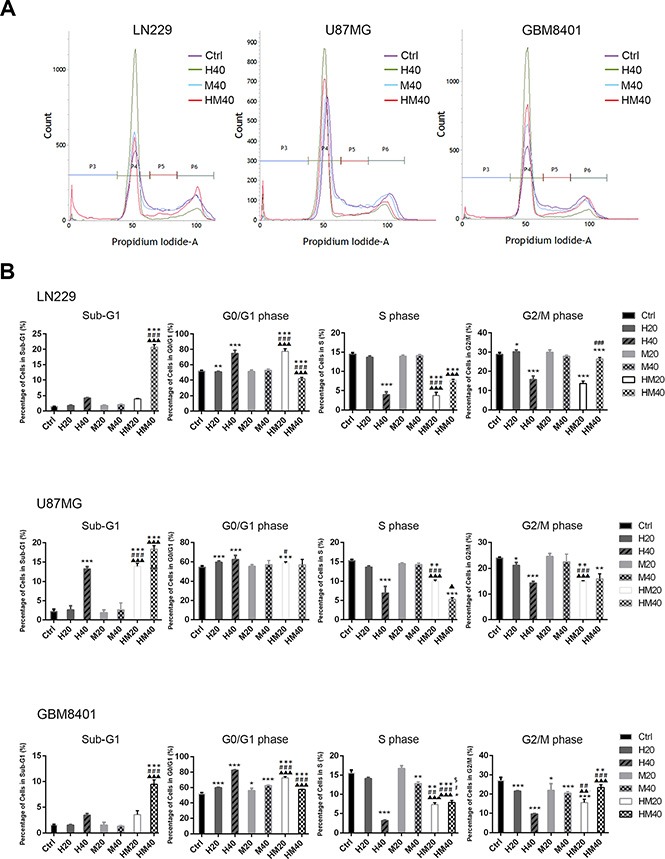
Cell cycle regulation by Hono, Mag and Hono-Mag combination treatment in human GBM cells (**A**) LN229, U87MG and GBM8401 glioma cells were treated with Hono, Mag or Hono-Mag (20–40 μM) for 24 hours individually. The cell cycle expression was then analyzed by FACS. (**B**) Quantitative analyses of cell populations in the sub-G1, G0/G1, S, and G2/M phases of the cell cycle were conducted using BD FACSuite analysis software. *p < 0.05; **p < 0.01; ***p < 0.001 compared to the control group. ^#^*p* < 0.05; ^##^*p* < 0.01; ^###^*p* < 0.001 compared to the same concentration of Hono-treated group. ^▲^*p* < 0.05; ^▲▲^*p* < 0.01; ^▲▲▲^*p* < 0.001 compared to the same concentration of Mag-treated group.

**Figure 3:**
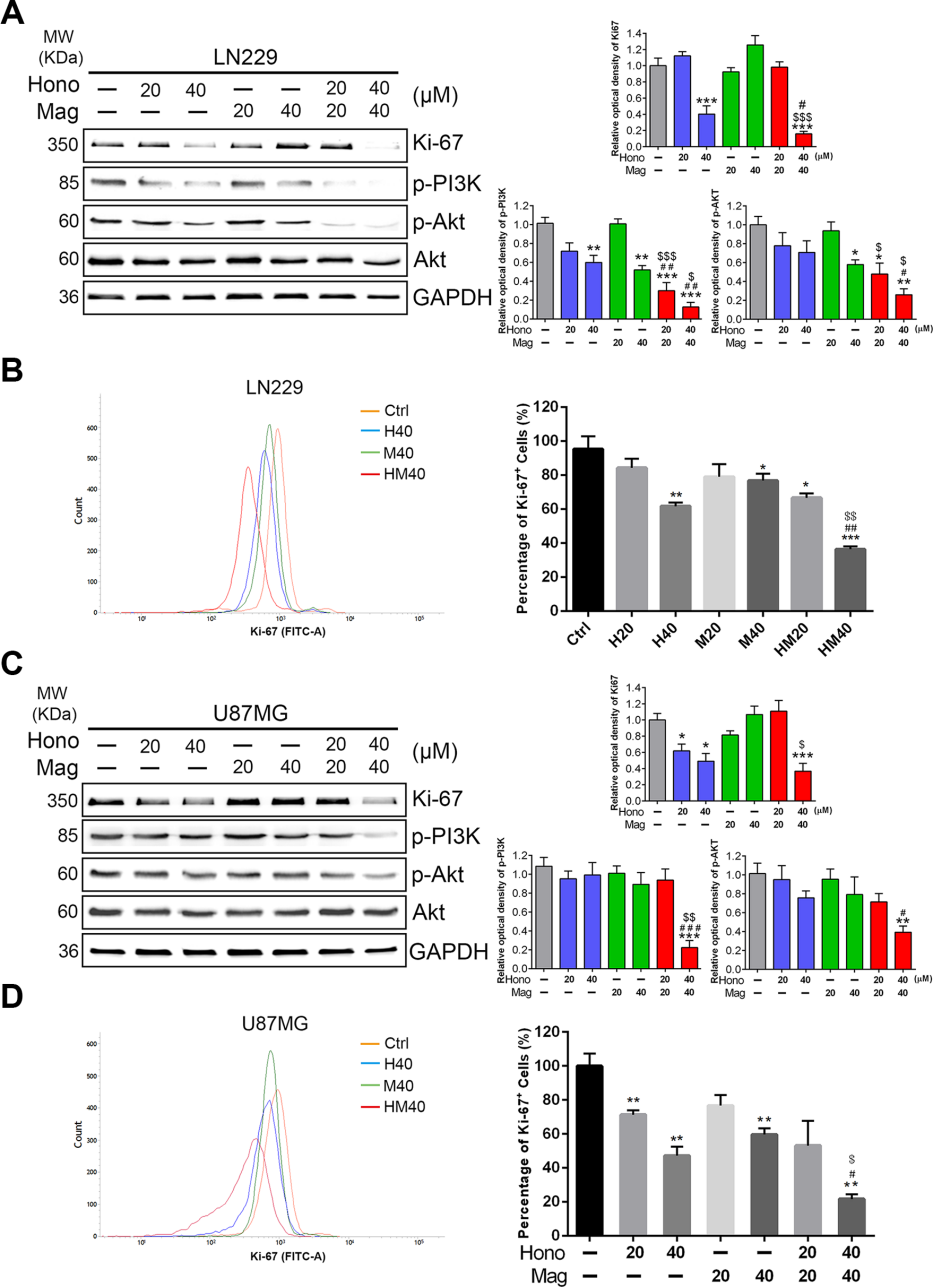
Hono-Mag combination treatment reduced the expression of proliferation-related protein in human GBM cells LN229 and U87MG glioma cells were treated with Hono, Mag or Hono-Mag (20–40 μM) for 24 h. The cell lysates of (**A**) LN229 and (**C**) U87MG were then analyzed for Ki-67, p-PI3K and p-Akt using Western blotting. GAPDH was used as the loading control. The right panels show the quantitative analyses. The protein expression of Ki-67 in (**B**) LN229 and (**D**) U87MG cells was analyzed using FACS. The right panels show the quantitative analyses. **p < 0.01; ***p < 0.001 compared to the control group. ^#^*p* < 0.05; ^##^*p* < 0.01 compared to the same concentration of Hono-treated group. ^$^*p* < 0.05; ^$$^*p* < 0.01;^$$$^*p* < 0.001 compared to the same concentration of Mag-treated group.

**Figure 4:**
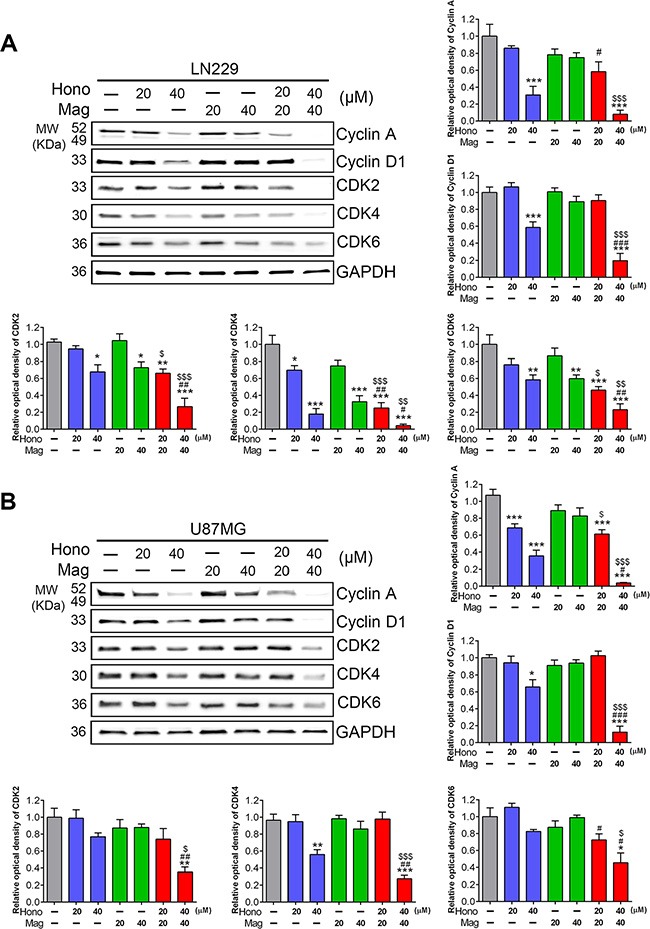
Hono-Mag treatment altered the expression of cell cycle-related proteins in human GBM cells LN229 and U87MG cells were treated with different concentrations of Hono, Mag or Hono-Mag for 24 hours. Protein expression levels of (**A**) LN229 and (**B**) U87MG were analyzed for cyclin A, cyclin D1, CDK2, CDK4 and CDK6 by Western blotting. GAPDH was used as an internal control. The panels show the quantitative analyses. *p < 0.05; **p < 0.01; ***p < 0.001 compared to the control group. ^#^*p* < 0.05; ^##^*p* < 0.01; ^###^*p* < 0.001 compared to the same concentration of Hono-treated group. ^▲^*p* < 0.05; ^▲▲^*p* < 0.01; ^▲▲▲^*p* < 0.001 compared to the same concentration of Mag-treated group.

To investigate the cell cycle regulatory mechanisms of Hono and Mag in cell growth inhibition, LN229, U87MG and GBM8401 cells were cultured with or without 40 μM Hono (H40), 40 μM Mag (M40), or a 40 μM Hono-Mag combination (HM40). As shown in Figure 2A, 2B, the Hono treatment resulted in concentration-dependent cell cycle arrest at the G0/G1 phase in human LN229, U87MG and GBM8401 cells. However, Mag-stimulated cell cycle arrest at the G0/G1 phase was only observed in GBM8401 cells. HM40 treatment increased the sub-G1 phase cell population and caused apoptosis. Besides, the Hono-Mag treatment reduced the protein expression of Ki-67 (Figure 3A, 3C) and Ki-67-positive cells in LN229 and U87MG cells (Figure 3B, 3D and Supplementary Figure 1). In addition, the HM20 and HM40 treatments decreased the PI3K and Akt in LN229 and U87MG cells (Figure 3A, 3C). In LN229 and U87MG glioma cells, the protein expression of cyclin A was decreased in the H40, HM20 and HM40 groups. Moreover, the expression of cyclin D1 was decreased under the H40 and the HM40 treatment (Figure 4A, 4B). Furthermore, the protein levels of CDK2, CDK4, and CDK6 declined significantly after HM20 and HM40 treatment compared to other groups in both U87MG and LN229 cells.

### The effect of hono and mag on apoptosis, autophage-associated proteins and MAPKS in human glioblastoma cells

**Figure 5:**
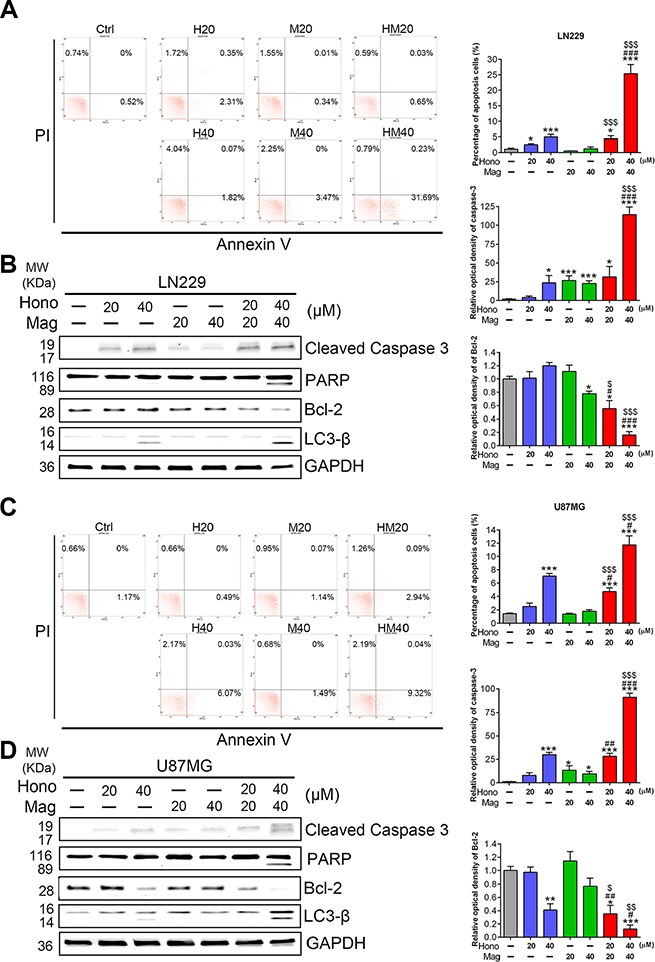
Hono-Mag promoted apoptosis and autophagy-associated proteins in GBM cells LN229 and U87MG cells were treated with Hono, Mag or Hono-Mag (20–40 μM) for 24 hours. The apoptotic cells of (**A**) LN229 and (**C**) U87MG were determined using Annexin V staining and detected by flow cytometry analysis. The right panels show the percentage of the apoptotic cells. The cell lysates of (**B**) LN229 and (**D**) U87MG cells were then analyzed for cleaved caspase 3, cleaved PARP, Bcl-2 and LC3β by Western blotting. GAPDH was used as the loading control. The right panels show the quantitative analyses of the proteins. *p < 0.05; ***p < 0.001 compared to the control group. ^#^*p* < 0.05; ^###^*p* < 0.001 compared to the same concentration of Hono-treated group. ^▲^*p* < 0.05; ^▲▲▲^*p* < 0.001 compared to the same concentration of Mag-treated group.

**Figure 6:**
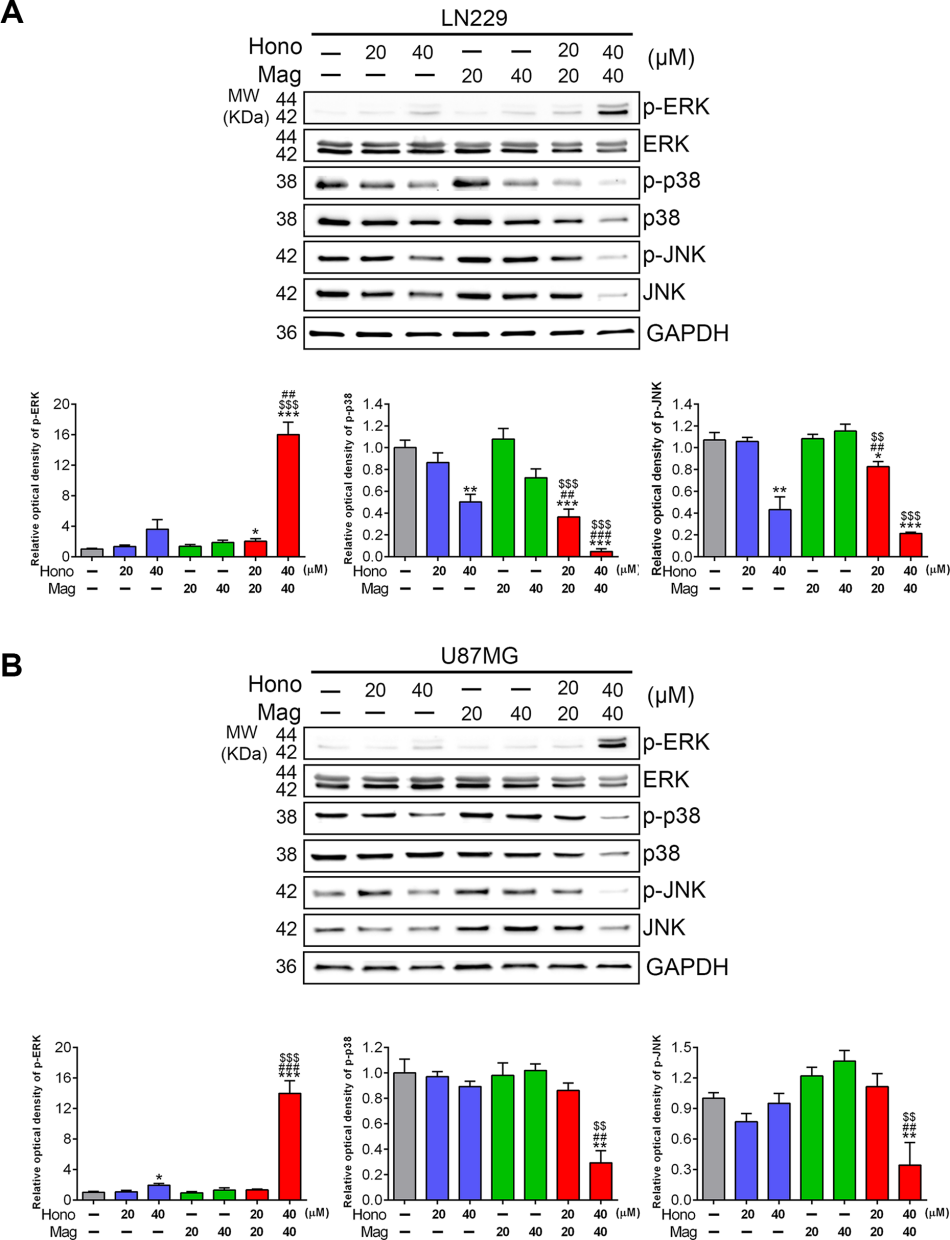
Hono-Mag down-regulated the activation of MAPKs in human GBM cells LN229 and U87MG were treated with Hono, Mag or Hono-Mag (20–40 μM) for 24 hours to confirm the activation of MAPKs. The expression levels of p-ERK, ERK, p-p38, p38, p-JNK, and JNK in (**A**) LN229 and (**B**) U87MG cells were detected using Western blotting. GAPDH was used as the loading control. The lower panels show the quantitative analyses of the proteins. *p < 0.05; **p < 0.01; ***p < 0.001 compared to the control group. ^##^*p* < 0.01; ^###^*p* < 0.001 compared to the same concentration of Hono-treated group. ^▲▲^*p* < 0.01; ^▲▲▲^*p* < 0.001 compared to the same concentration of Mag-treated group.

**Figure 7:**
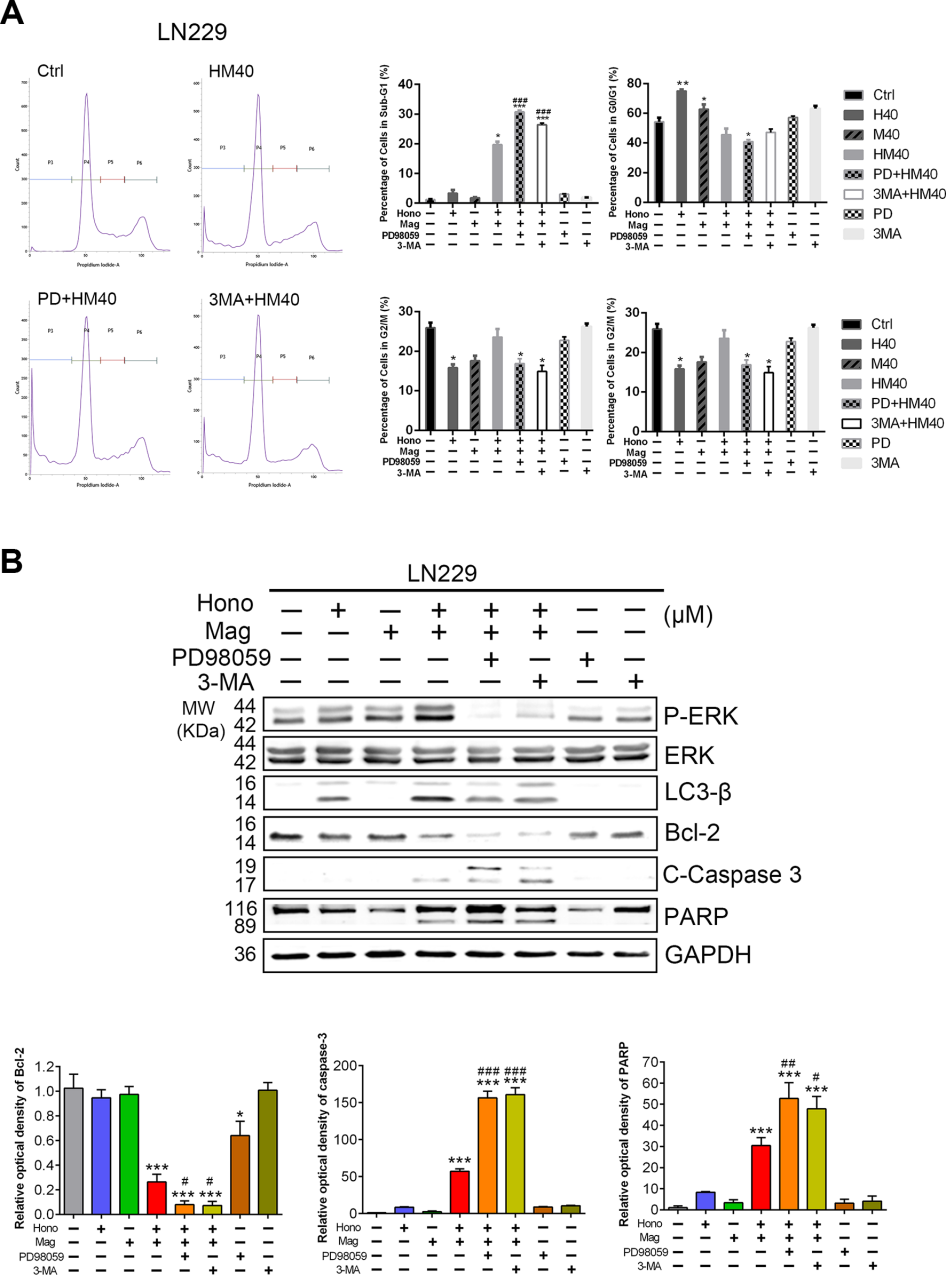
Suppression of p-ERK or autophagy enhanced Hono-Mag-stimulated apoptosis in human GBM Cells The 40 μM of Hono, Mag, or Hono-Mag groups were combined with PD98059 or 3-MA in LN229 cells. After 24 hours of treatment, these cells were analyzed for populations in different stages of the cell cycle and protein expression. (**A**) The percentage of the cell population in the sub-G1, G0/G1, S, and G2/M phases of the cell cycle was determined using BD FACSuite analysis software. The right panels show quantitative analyses. (**B**) The protein expression levels of p-ERK, ERK, LC3β, Bcl-2, cleaved caspase 3 and PARP in LN229 cells were detected by Western blotting. GAPDH was used as the loading control. The lower panels show the quantitative analyses of the proteins. *p < 0.05; ***p < 0.001 compared to the control group. ^#^*p* < 0.05; ^##^*p* < 0.01; ^###^*p* < 0.001 compared to the same concentration of Hono-Mag treatment group.

Annexin V and PI staining showed that HM40 treatment promoted apoptosis in U87MG and LN229 cells (Figure 5A, 5C). To further reveal the underlying mechanism of HM40-induced apoptosis, apoptotic-related proteins were examined. The cleaved caspase-8, caspase-9 (Supplementary Figure 2), caspase-3 and PARP (Figure 5B, 5D) were increased after HM40 treatment in LN229 and U87MG cells. In addition, the anti-apoptosis protein Bcl-2 was decreased after HM40 treatment (Figure 5B, 5D). Moreover, the expression of LC3-βII, an autophagy marker, was observed after HM40 treatment. As shown in Figure 6A, the HM40 treatment decreased the protein expression of p-p38 and p-JNK but increased p-ERK in LN229 cells (Figure 6A). A similar effect was observed in U87MG cells (Figure 6B). To investigate the roles of p-ERK and autophagy induced by HM40 treatment, the HM40 treatment groups were pretreated with ERK inhibitor (PD98059) or autophagy inhibitor (3-MA). Both PD98059 and 3-MA increased the HM40-induced cell population at the sub-G1 phase (Figure 7A). Furthermore, both PD98059 and 3-MA reduced the Hono-Mag-increased p-ERK, LC3βII, and Bcl-2, but enhanced the expression of cleaved caspase-3 and cleaved PARP in LN229 cells (Figure 7B). The HM40 treatments with PD98059 or 3-MA significantly enhanced Hono-Mag-induced cell apoptosis, suggesting that ERK activation and autophagy played rescue roles.

### The effect of hono-mag combination treatment on orthotropic human glioblastoma xenograft mouse models

**Figure 8:**
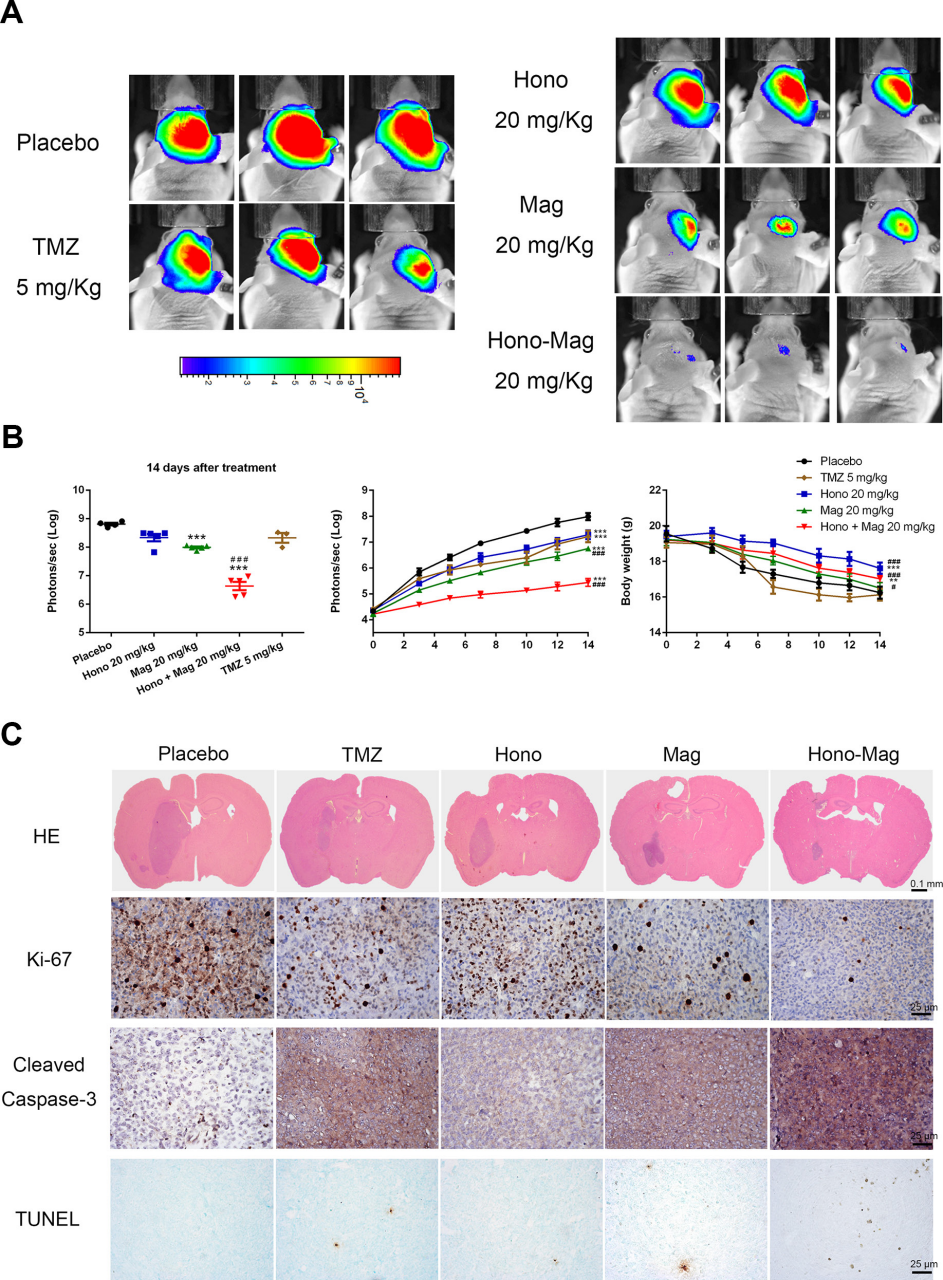
Hono-Mag treatment reduced tumor progression in orthotropic human GBM xenograft mouse models (**A**) The *in vivo* bioluminescent imaging data were analyzed in different groups via the IVIS system. The lower panels show the quantitative analyses of the tumor progression. ***p < 0.001 compared to the placebo group. ^###^*p* < 0.001 compared to the TMZ-treated group. (**B**) The body weight was measured during drug treatment. **p < 0.05; ***p < 0.001 compared to the placebo group. ^#^*p* < 0.05; ^###^*p* < 0.001 compared to the TMZ-treated group. (**C**) Representative images of histological staining and immunohistochemical stainings in xenograft orthotropic brain tumors. Protein levels of Ki-67, cleaved caspase-3 and TUNEL assay were performed. Bar, 25 μm.

LN229 human glioblastoma cells, labeled with luciferase, were implanted intracranially. After treatment with Hono-Mag, tumor growth was significantly decreased compared to the Hono, Mag, TMZ and vehicle control groups (Figure 8A). The TMZ treatment only partially restricted tumor growth. Besides, the body weight in Hono and Hono-Mag groups was higher than in the TMZ and PBS groups (Figure 8B). HE and IHC staining revealed that Hono-Mag combination treatment not only inhibited Ki-67 expression and tumor growth but also activated the caspase-3 to enhance cells apoptosis in the tumors of nude mice (Figure 8C). Thus, the Hono-Mag combination treatment inhibited the progression and survival of glioblastoma cells both *in vitro* and *in vivo*.

## DISCUSSION

Both Mag and Hono have been reported to exert anti-cancer effects via inhibiting proliferation, inducing apoptosis, countering metastasis, suppressing angiogenesis, and reversing multidrug resistance [18–20]. Until now, no studies had explored the effect of Mag-Hono combination treatment on glioblastoma; however, the present study found that both *in vitro* and *in vivo*, Mag-Hono combination treatment showed more effective in anti-proliferation and apoptosis than single treatment in GBM cells.

In glioma therapy, Mag treatment inhibits human U373 cell proliferation and induces apoptosis through p21/Cip1 and p27/Kip1 induction *in vitro* [10]. Hono also caused cell cycle arrest by decreasing the expression of cell cycle-related proteins, such as cyclin D1, CDK4 and CDK6 in PC-3 prostate cancer cells [21]. However, no studies affirmatively supported the combination of these two compounds or explored the underlying mechanism in glioma therapy. Hono treatment decreases the phosphorylation of Akt, signal transducer and activator of transcription 3 and ERK2 in colon cancer, malignant melanoma, and SVR endothelial tumors [21–23]. In addition, Mag inhibits EGFR, PI3K, and Akt activation in human prostate cancer [24]. The activation of PI3K and Akt plays a critical role in the survival and progression of GBM cells [25, 26]. Hyperactivation of PI3K and Akt may cause poor prognosis and chemotherapeutic/radiotherapeutic resistance [27]. The phosphorylation of Akt inhibits GSK3, contributing to the stabilization of cyclin D1 [28]. In LN229 and U87MG cells, the HM20 and HM40 treatments decreased the activation of PI3K, Akt and Akt-associated cell cycle-related proteins, such as cyclin D1 and cyclin A. In addition, Hono-Mag combination treatment inactivated CDK4, CDK6, Akt and decreased the proliferation through cell cycle arresting at the G0/G1 phase in GBM cells.

Apoptosis and autophagy were induced by 40 μM Hono-Mag combination treatment. Hono-Mag-induced Akt dephosphorylation may activate the pro-apoptotic Bcl-2 family member Bad and decrease the expression of the anti-apoptotic proteins Bcl-XL and Bcl-2, which prevent the release of cytochrome c from mitochondria [30, 31]. According to our results, the HM40 treatment decreased Bcl-2 expression and caused the cleavage of caspase-8, caspase-9, caspase-3 and PARP, which contributed to apoptosis through both intrinsic and extrinsic pathway in LN229 and U87MG cells. JNK and p38 have been reported to have either pro- or anti-apoptotic functions, depending on the cell types and microenvironments [32–34]. The inhibition of p38 triggers the TNF-mediated and caspase-dependent apoptotic pathway in human lymphoma U937 cells and increases sensitivity to the cytotoxic effect of TMZ in human glioma U87MG cells [35, 36]. In addition, the inhibition of JNK suppresses the activation of Akt and its downstream mediators, GSK-3β and Bad, but potentiates TMZ-induced cytotoxicity in U87 glioma cells [37]. In the present study, both phosphorylated p38 and JNK decreased after Hono-Mag combination treatment. These results revealed that apoptosis induced by Hono-Mag treatment may be caused by the attenuation of Akt, p38 MAPK and JNK in U87MG and LN229 cells.

Autophagy, a process conventionally considered as a survival mechanism, is increasingly being applied to facilitate cancer cell death by chemotherapy [38, 39]. Several studies indicate that both Mag and Hono are involved in autophagy induction [17, 40]. In human umbilical cord vein endothelial cells and prostate cancer cells, Mag-caused autophagy inhibited cell proliferation, migration, invasion and tube formation [13]. In human non-small-cell lung cancer cells, the combination of Hono and autophagy inhibitor inhibited the Hono-stimulated autophagy and induced apoptosis in a caspase-dependent manner [41]. Thus, an effective treatment may involve a combined and coherent action of apoptosis as well as non-apoptotic programs to minimize the chance of relapse [42]. Here, Hono-Mag-induced autophagy was blocked by both ERK and autophagy inhibitors. Recently, Hono was reported to induce autophagy and suppress cell migration through activating the PI3K/Akt/mTOR and endoplasmic reticular stress/ERK signaling pathways in neuroblastoma cells [43]. However, the attenuation of Hono-induced cell autophagy also down-regulates cell apoptosis *in vitro* [43]. According to our results, Hono-Mag treatment combined with PD98059 or 3-MA, significantly enhanced Hono-Mag-stimulated apoptosis in LN229 cells. Therefore, the induction of ERK phosphorylation resulting from Hono-Mag treatment played a critical role in rescuing apoptosis.

The first-line chemotherapeutic drug for GBM is the alkylating agent TMZ. Through oral absorption, TMZ is converted to an alkylating methyldiazonium cation, leading to cell death by breaking the DNA double strand [44, 45]. Human glioblastoma cell line LN229 has been reported to be temozolomide resistant [46, 47]. In the orthotopic xenograft mouse model, compared with the TMZ, Hono, and Mag treatment groups, the Hono-Mag combination treatment induced apoptosis and reduced the growth and progression of GBM tumors. These results indicated that the Hono-Mag combination has additional potential as an effective anti-tumor agent to target TMZ-resistant cells. Moreover, Hono-Mag combination treatment significantly maintained the body weight of mice comparing with the TMZ and PBS groups. Taking into account the ability to cross the BBB and the synergistic effect of Hono and Mag on glioma cell growth and progression inhibition, the Hono-Mag combination treatment may be applied as an adjuvant therapy to improve the therapeutic efficacy of GBM treatment.

## MATERIALS AND METHODS

### Cell culture

Human U87MG and LN229 glioblastoma cell lines were purchased from the American Type Culture Collection (Rockville, MD). LN229-Luc2 cells were derived via the stable transfection of pLuc2-iRFP and selected with a FACS Aria Fusion Sorter. The human glioblastoma multiforme (GBM8401) cell line was provided by Dr. Hueng from the National Defense Medical Center, Taiwan. The cells were grown in Dulbecco's Modified Eagle's Medium (DMEM) containing 10% fetal bovine serum and 100 IU/ml penicillin and streptomycin (pH 7.4) (all obtained from Gibco) in a humidified atmosphere of 5% CO_2_ at 37°C.

### Drugs

The 2.3.3-[4, 5-dimethylthiazol-2-yl]-2,5-diphenyltetrazolium bromide (MTT) used in this study was obtained from Sigma-Aldrich (St. Louis, MO). Propidium iodide (PI), honokiol, magnolol, PD98059 and 3-methyladenine (3-MA) were purchased from Sigma-Aldrich (Sigma). Temozolomide (TMZ) was purchased from MedChem Express (MCE).

### Cell survival assays

LN229, U87MG and GBM8401 glioblastoma cells were plated at 2 × 10^4^ cells per well in a 24-well plate. Different concentrations of drugs or vehicle control were then added to the culture medium for 24 hours. After the cells were washed with phosphate-buffered saline (PBS) (137 mM NaCl, 2.7 mM KCl, 1.5 mM KH_2_PO_4_, 8 mM Na_2_HPO_4_, and pH 7.4), 0.5 mg/ml of MTT was added and incubation continued for another 4 hours. The cells were then lysed with DMSO (Sigma). Absorbance at 590 nm was measured.

### Flow cytometric analysis

The cell cycle, apoptosis assays and expression of Ki-67 were prepared by seeding 2 × 10^5^ LN229, U87MG or GBM8401 glioma cells in 6-well plates. After cell attachment, growth medium either with or without different concentrations of Hono and Mag was added for 24 hours. To examine the expression of Ki-67, the cells were fixed and permeabilized at room temperature. Ki-67 primary antibody (Genetex) was incubated for 40 minutes. After PBS wash, the secondary antibodies conjugated to goat anti-rabbit antibody (Alexa Fluor 488, 1:500 Molecular Probes) were incubated for 40 minutes at room temperature. For cell cycle assays, cells were fixed in ethanol and stained with PI. For apoptosis assays, cells were harvested and processed for Annexin V staining according to the manufacturer (Invitrogen). The cells were washed with binding buffer [4-(2-hydroxyethyl)-1-piperazineethanesulfonic acid, 140 mmol/l NaCl, and 5 mmol/l CaCl_2_ at pH 7.4] and stained with anti-Annexin V antibody (FITC) and then counterstained with PI for 15 minutes at room temperature. The results were measured using a FACS Verse laser flow cytometric analysis system (Becton Dickinson). Ten thousand cells were analyzed for each sample.

### Western blotting

After the various treatments, the glioma cells were washed once with PBS and then homogenized in lysis buffer (10 mM EGTA, 2 mM MgCl_2_, 60 mM PIPES, 25 mM HEPES, 0.15% Triton X-100, 1 μg/ml of pepstatin A, 1 μg/ml of leupeptin, 1 mM NaF, and 1 mM phenylmethylsulfonyl fluoride). Protein samples (60 μg per lane) were electrophoresed on a 10% SDS polyacrylamide gel and transferred to a nitrocellulose membrane (Bio-Rad). Strips from the membrane were blocked with 5% non-fat milk in Tris-buffered saline, pH 8.2, containing 0.1% Tween (TBS-Tween) and incubated overnight at 4°C with a 1:500 dilution of rabbit antibodies against phosphorylated phosphoinositide 3-kinase (p-PI3K), cyclin-dependent kinase 2 (CDK2), CDK4, CDK6, phosphorylated JNK (p-JNK), JNK, phosphorylated ERK (p-ERK), ERK, phosphorylated p38 (p-p38), p38, Poly ADP ribose polymerase (PARP), cleaved caspase-3, cleaved caspase-8, cleaved caspase-9 (Cell Signaling), phosphorylated Akt (p-Akt) (Epitomics), Akt, cyclin A, cyclin D1 (Abcam), Bcl-2, GAPDH (BioVision), and Ki-67 (Genetex). After washes, the strips were incubated with a 1:5000 dilution of HRP-conjugated anti-rabbit IgG antibodies (Promega). Then, the blots were reacted in ECL substrate developing solution (Bio-Rad). The density of the bands on the nitrocellulose membrane was quantified by densitometry using Gel Pro 3.1 (Media Cybernetics), taking the density of the control sample as 100% and expressing the density of the test sample relative to the expression of the internal control as a relative value. Phosphorylated proteins were normalized to the total protein first.

### Immunocytochemistry

U87MG and LN229 cells were grown on coverslips and fixed in cold 4% paraformaldehyde for 30 minutes at room temperature. The fixed cells were permeabilized with 0.1% Triton X-100 at room temperature and blocked with 3% bovine serum albumin (BSA). Ki-67 (Genetex) primary antibody was incubated overnight at 4°C. After PBS washing three times, the conjugated goat anti-rabbit IgG secondary antibodies (Alexa Fluor 488, 1:500 Molecular Probes) were incubated for 1 hour at room temperature. The samples were mounted onto slides and visualized using a confocal microscope (Zeiss).

### Animal xenograft model

BALB/cAnN.Cg-Foxn1nu/CrlNarl nude mice (20–25 g) were anesthetized with O_2_/isoflurane mixture. Then, 10^5^ LN229-Luc2 cells were implanted in the right cerebral hemisphere of the mice. Gliomas were permitted to grow in the murine brain. Five days after implantation, mice were randomly assigned to five groups (*n* = 5) that received vehicle control, Hono, Mag, Hono-Mag or TMZ. Hono, Mag and Hono-Mag were administered via intraperitoneal injection of 20 mg/kg/day for 14 days. TMZ was administered via oral gavage of 5 mg/kg/day for 7 days. Animals were euthanized after 14 days. The bioluminescence intensity of implanted tumors was monitored using the non-invasive*In Vivo* Imaging System (IVIS) three times per week. The body weight of the mice was measured three times per week.

### Histological and immunohistochemistry examination

Orthotopic tumors were excised, rinsed twice in PBS and fixed in 4% paraformaldehyde for 48 hours. Tissues were frozen and sliced into 5-μm-thick sections. Routine hematoxylin and eosin (HE) staining was performed to facilitate histological evaluation. The expression of Ki-67, cleaved caspase-3 and BrdU in brain tumors of the nude mice was detected by immunohistochemistry. Ki-67 (Genetex) and cleaved caspase-3 (Cell signaling) primary antibodies and secondary goat anti-rabbit antibodies (Jackson Laboratories) were used. The apoptotic cells were detected via Apo-BrdU-IHC *In Situ* DNA Fragmentation assay kit (BioVision). The expression of Ki-67, cleaved caspase-3 and BrdU was observed in 10 random fields for each group.

### Statistical analysis

All experiments were performed at least three times, and the results were expressed as the mean ± SEM for the total number of experiments. Differences between means were assessed using the Kruskal-Wallis test. The Mann-Whitney test was used for post hoc analysis. Statistical significance was set at *p* < 0.05.

## SUPPLEMENTARY MATERIALS FIGURES AND TABLES


