# Supplementary material for: Magnolol and honokiol exert a synergistic anti-tumor effect through autophagy and apoptosis in human glioblastomas
Source: Oncotarget. 2016 Apr 11;7(20):29116–30. doi: 10.18632/oncotarget.8674 (PMC5045382; doi:10.18632/oncotarget.8674)
Supplement: Supplementary file 1 [file oncotarget-07-29116-s001.pdf]

# Magnolol and honokiol exert a synergistic anti-tumor effect through autophagy and apoptosis in human glioblastomas

## Supplementary Materials

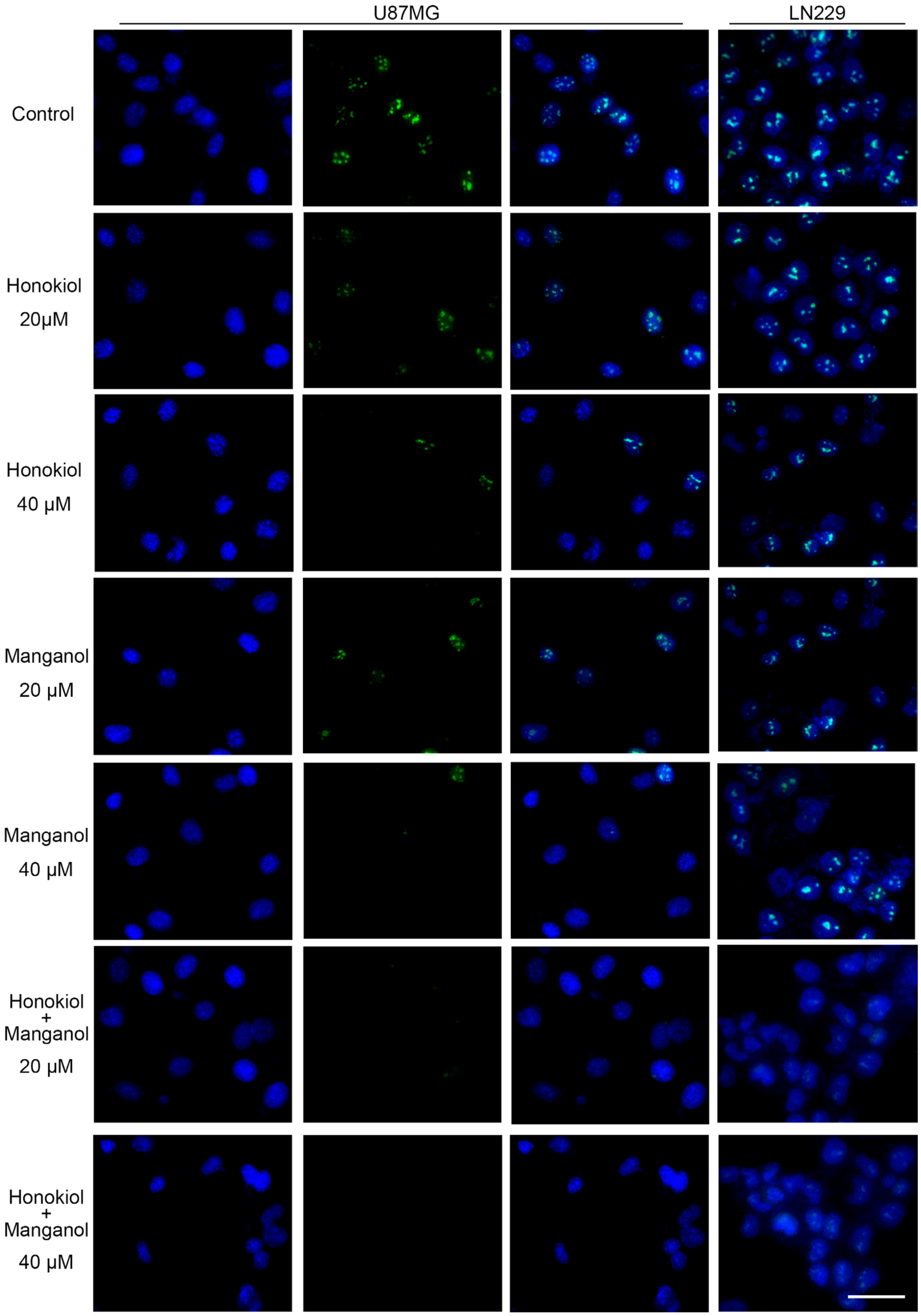


**Supplementary Figure S1: The images of Ki-67 expression in U87MG and LN229 cells by immunocytochemical stainings.** The combination treatment of Hono and Mag substantially decreased the expression of Ki-67 in LN229 and U87MG cells as compared to the control group, Hono-alone group, or Mag-alone group. Bar, 20 μm.

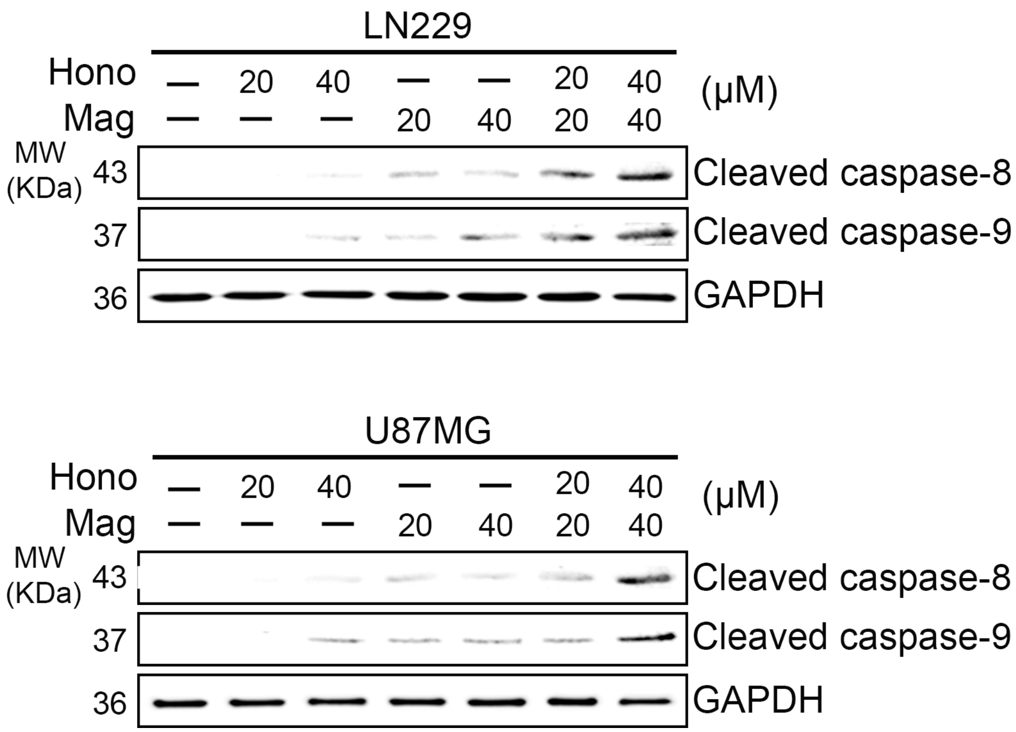


**Supplementary Figure S2: Hono-Mag increased the protein expression of cleaved caspase 8 and cleaved caspase 9 in human GBM cells.** The cell lysates of LN229 and U87MG cells were analyzed for cleaved caspase-8 and cleaved caspase-9 by Western blotting. GAPDH was used as the loading control.
